# Recombinant Factor IX Fc Fusion Protein Maintains Full Procoagulant Properties and Exhibits Prolonged Efficacy in Hemophilia B Mice

**DOI:** 10.1371/journal.pone.0148255

**Published:** 2016-02-03

**Authors:** Garabet G. Toby, Tongyao Liu, Yang Buyue, Xin Zhang, Alan J. Bitonti, Glenn F. Pierce, Jurg M. Sommer, Haiyan Jiang, Robert T. Peters

**Affiliations:** Biogen, Cambridge, MA, United States of America; Emory University School of Medicine, UNITED STATES

## Abstract

**Introduction:**

Hemophilia B is an inherited X chromosome–linked disorder characterized by impaired blood clotting owing to the absence of functional coagulation factor IX. Due to the relatively short half-life of factor IX, patients with hemophilia B require frequent factor IX infusions to maintain prophylaxis. We have developed a recombinant factor IX (rFIX) fused to the Fc region of IgG (rFIXFc) with an extended half-life in animals and humans.

**Materials and Methods:**

Procoagulant properties of rFIXFc and rFIX (BENEFIX^®^) were compared to determine the effect of the Fc region on rFIXFc hemostatic function. Specifically, we assessed rFIXFc activation, intermolecular interactions within the Xase complex, inactivation by antithrombin III (AT) and thrombin generation potential compared with rFIX. We also assessed the acute and prophylactic efficacy profiles of rFIXFc and rFIX *in vivo* in hemophilia B mouse bleeding models.

**Results and Conclusions:**

The activation by factor XIa or factor VIIa/tissue factor, inhibition by AT, interaction profiles with phospholipids, affinities for factor VIIIa within the context of the Xase complex, and thrombin generation profiles were similar for rFIXFc and rFIX. Xase complexes formed with either molecule exhibited similar kinetic profiles for factor Xa generation. In acute efficacy models, mice infused with rFIXFc or rFIX were equally protected from bleeding. However, in prophylactic efficacy models, protection from bleeding was maintained approximately three times longer in rFIXFc-dosed mice than in those given rFIX; this prolonged efficacy correlates with the previously observed half-life extension. We conclude that rFIXFc retains critical FIX procoagulant attributes and that the extension in rFIXFc half-life translates into prolonged efficacy in hemophilia B mice.

## Introduction

Blood clotting in response to vessel and capillary damage is essential for normal hemostasis. Hemophilia B is an inherited X chromosome–linked disorder characterized by the inability to clot blood and is due to the absence or diminished levels of coagulation factor IX (FIX) [1]. In its severe form (FIX activity <1%), patients may experience bleeding either spontaneously or following an injury; over time, repeated bleeding episodes in the joints and muscles can result in severe arthropathy. The current standard of care for patients with hemophilia B is replacement therapy through infusion of recombinant or highly purified plasma FIX concentrates either on demand to treat bleeding or prophylactically to prevent bleeding [1,2]. In on-demand therapy, patients receive FIX concentrates in response to injury, hemorrhage, or prior to surgery and may require multiple infusions to achieve and maintain protective factor levels. In prophylaxis, a preferred regimen for severe hemophilia B, patients infuse replacement factor (rFIX or plasma-derived FIX) two to three times per week with the goal of maintaining adequate levels of FIX to prevent bleeding [3,4]. The frequency of infusions required for prophylactic treatments (generally 2–3 times per week) is a limiting aspect of treatment and due in part to the relatively short half-life of conventional FIX products [5–7].

To extend the circulating half-life of FIX and, therefore, reduce the dosing frequency for hemophilia B therapy, we have developed a recombinant factor IX Fc fusion protein (rFIXFc) that is composed of a single molecule of rFIX covalently fused to the Fc domain of human immunoglobulin G1 (IgG1) with no intervening linker sequence [8,9]. Fc fusion leverages an endogenous pathway utilized by IgG to extend the half-life of rFIXFc by enabling interactions intracellularly with the neonatal Fc receptor (FcRn). IgG catabolism is normally prevented by FcRn in vascular endothelium and monocytes, which contributes to the long half-life of antibodies [10–14]. We previously reported that in this monomeric configuration, the elimination half-life of rFIXFc is approximately 3- to 4-fold longer than that of recombinant FIX (rFIX) in multiple animal models including normal mice, rats and monkeys and in FIX-deficient mice and dogs [8]. In addition, a prolonged half-life of rFIXFc in comparison with rFIX was also observed in humans in phase 3 clinical trials (geometric mean, 82.1 hours for rFIXFc vs 33.8 hours for rFIX; *P* <0.001 in a phase 3 trial) [15].

In the present study, we characterized the effect of the Fc moiety on the procoagulant function of rFIXFc compared with that of rFIX. Specifically, we assessed rFIXFc activation, intermolecular interactions within the Xase complex, inactivation by AT, and the thrombin generation potential relative to rFIX. We also compared the acute efficacy of rFIXFc and rFIX *in vivo* to resolve bleeding in mouse models. Finally, we assessed the prophylactic efficacy profile of both proteins *in vivo* in mouse bleeding models to determine if the previously observed half-life extension translates into prolonged protection from bleeding.

## Materials and Methods

### Ethics statement

This study was carried out in strict accordance with the recommendations in the Guide for the Care and Use of Laboratory Animals of the National Institutes of Health. All study protocols were reviewed and approved by the Institutional Animal Care and Use Committee (IACUC) of Biogen (Permit Numbers: 01–10 and 02–10) in a vivarium accredited by the Association for Assessment and Accreditation of Laboratory Animal Care International (AAALAC; file #1330). All surgery was performed under anesthesia, and all efforts were made to minimize suffering [16]. More specifically, at the desired time point, the mice were anesthetized with a Ketamine/Dexmedetomidine/Buprenex cocktail. This cocktail provided a Ketamine dose of 50 mg/kg, a Dexmedetomidine dose of 0.5 mg/kg, and a Buprenex dose of 0.1 mg/kg (when injected at 5 mL/kg of body weight intraperitoneally). For the mouse that could not reach adequate anesthetic depth, another 50 μL of 10 mg/mL of Ketamine, approximately 20 mg/kg (mix 1 mL of 100 mg/mL of Ketamine solution with 9 mL of sterile saline) were injected. After tail vein transection, the mouse was returned to its individual cage with white paper bedding placed on top of a heating pad. In the following 11 hours and then overnight at 24 hours, the study animals were monitored hourly and euthanized immediately with a lethal dose of CO_2_ when they reached moribund state (defined as being recumbent and unresponsive to external stimuli) [16]. At the last time point before going overnight, the remaining mice received 0.1 mg/kg Buprenex for pain relief. All mice were euthanized at 24 hours after tail vein transection.

### Reagents

rFIXFc was manufactured at Swedish Orphan Biovitrum AB (Stockholm, Sweden) and Biogen (Cambridge, MA, USA). The rFIX used in these studies was BENEFIX^®^ (coagulation factor IX, recombinant; Pfizer Inc. [Philadelphia, PA, USA]) and the FVIII used was Kogenate FS^®^ (recombinant antihemophilic factor; Bayer HealthCare [Tarrytown, NY, USA]). FIXa, FVIIa, FX, FXa, FXIa, AT and α-thrombin were purchased from Haematologic Technologies (Essex Junction, VT, USA); lipidated tissue factor from Sekisui Diagnostics (formerly American Diagnostica Inc.; Stamford, CT, USA); heparin was from Elkins-Sinn Inc (Cherry Hill, NJ, USA); and the recombinant hirudin, FXa fluorogenic substrate and chromogenic substrates for FXa and FIXa were from Centerchem, Inc. (Norwalk, CT, USA). The anti-CD62 IgG1 antibody specific for membrane glycoprotein P-selectin was purchased from BD Biosciences (Franklin Lakes, NJ, USA) and IgG1 from eBioScience (San Diego, CA, USA). Unactivated partial thromboplastin time reagent used as the cephalin source was purchased from Bio/Data Corp. (Horsham, PA, USA). The phospholipids used for vesicle preparation (L-α-phosphatidylserine [PS] and L-α-phosphatidylcholine [PC]) were purchased from Avanti Polar Lipids (Alabaster, AL, USA). Fresh platelets from normal volunteers were purchased from AllCells, LLC (Emeryville, CA, USA). The mini-extruder for phospholipid vesicle generation was obtained from Avanti Polar Lipids (Alabaster, AL, USA). Absorbance and fluorescence were read on a BioTek Synergy 2 plate reader with Gen5™ software (Winooski, VT, USA). A COULTER^®^ EPICS^®^ XL-MCL™ Flow Cytometer (Miami, FL, USA) was used for all flow cytometric measurements.

### Biochemical characterization of rFIXFc

Complete experimental details of each of the biochemical assay methods are included in the Methods in S1 Text. Briefly, activation of rFIXFc and rFIX was evaluated after incubation at 37°C with FXIa for up to 5 min or with FVIIa/TF for up to 15 min. Activated mixtures were placed on ice, stored at –20°C, and assessed by sodium dodecyl sulfate–polyacrylamide gel electrophoresis (SDS-PAGE) analysis. Inhibition of rFIXaFc by AT was evaluated by incubating rFIXaFc or rFIXa with various concentrations of AT at room temperature (RT) for 30 minutes. The residual activity of rFIXaFc or rFIXa toward FX was assessed in a FXa generation assay. This assay was conducted purposely in the absence of FVIIIa to control reaction rates and facilitate data collection on a measureable timescale. Formation of the FVIIIa-FIXa (Xase) complex was evaluated for rFIXaFc and rFIXa using a chromogenic substrate to monitor FXa activity. The affinity of rFIXaFc and rFIXa for FVIIIa was determined using three different phospholipid sources (25% PS/75% PC, cephalin or platelets). In each case, rFIXaFc or rFIXa was incubated with the phospholipid surface and thrombin-activated FVIIIa for 10 minutes at RT to allow for the formation of the Xase complex. FXa generation was determined using a chromogenic substrate (PS/PC or cephalin). For the platelet assay, platelets were added to the reaction mix along with a fluorogenic substrate and fluorescence intensity was monitored over time.

### Enzyme kinetics of FX activation by FIXaFc/FVIIIa

The Xase complex activity was assayed for both FXIa- and FVIIa/TF-activated rFIXFc and rFIX. The affinity (K_M_) and V_*max*_ of Xase complexes formed by either rFIXaFc or rFIXa and FVIIIa were determined by measuring the FXa generation rates in reactions containing varying concentrations of FX (4 nM-4 μM). Briefly, FX was diluted in 50 mM Tris (pH 7.4), 100 mM NaCl, 5 mM CaCl_2_ and 0.2% BSA. As described in the Methods in S1 Text, FVIII (2 nM) was activated by thrombin and mixed with either rFIXaFc or rFIXa (1 nM) in the presence of hirudin and phospholipids to form the Xase complex. The Xase complex formed by either rFIXaFc or rFIXa was added to the various FX dilutions, and the conversion of FX into FXa was monitored in the presence of an FXa substrate. Rates of FXa generation were determined and the data were fitted to determine K_*M*_ and *V*_*max*_, as previously described [17].

### rFIXaFc levels in rFIXFc

Varying concentrations (1000, 500, 250 and 125 nM) of non-activated rFIXFc or rFIX were incubated with 2 nM α-thrombin–activated FVIIIa for 10 minutes to form the Xase complex. In parallel, 2 nM FVIIIa and 1 nM FXIa-activated rFIXFc or rFIX were incubated with cephalin to serve as positive controls for rFIXa activity. Rates of FXa generation were determined (as described in the Methods in S1 Text).

### Preparation of platelets and phospholipid vesicles

Fresh peripheral blood platelets (5 × 10^8^ platelets/mL) were processed immediately upon receipt, washed with calcium-free phosphate-buffered saline and the resultant suspension was centrifuged at 3000 rpm for 10 minutes. For activation, platelets were incubated at 37°C for 10 minutes with SFLLRN peptide (50 μg/mL). The activation of platelets was confirmed by a fluorescence-activated cell sorter (FACS) using CD62 and IgG1 isotype antibodies. FXa generation assays with non-activated platelets contained 2 × 10^8^ cells/mL, whereas assays with activated platelets contained 1 × 10^8^cells/mL to control the rate of FXa generation. Phospholipid vesicles (20 mM stock) consisting of 25% PS/75% PC were prepared using the Avanti^®^ Mini-Extruder (0.1-μm pore size membrane) according to the manufacturer’s instructions and stored at 4°C.

### Thrombin generation assay

FIX-deficient plasma was obtained from George King Bio-Medical, Inc (Overland Park, KS). Plasma-derived factor IXa and human thrombin were purchased from Haematologic Technologies (Essex Junction, Vermont). Thrombin activity was determined by the calibrated automated thrombogram (CAT) method described by Hemker et al. using the standard assay protocol and reagents from Thrombinoscope^®^ (Stago, Parsippany, NJ) [18]. Final concentrations of reagents were 1 pM tissue factor and 4 μM phospholipids for assay wells, or 630 nM thrombin calibrator for calibration wells. When thrombin generation was triggered with factor IXa, increasing concentrations (0–100 pM) of plasma-derived factor IXa, followed by 5 nM human thrombin, were added to FIX-deficient plasma in the presence or absence of factor IX variants 3 min prior to recalcification.

### Ex vivo activity in hemophilia B mice

Male hemophilia B mice were given 50 IU/kg of rFIXFc or 100 IU/kg of rFIX by intravenous injection and euthanized prior to blood collection. Whole blood was collected from the vena cava of treated animals into citrate at 5 minutes and at 24, 72, 96, 120, 168 and 216 hours after rFIXFc dosing (*n* = 8) or at 5 minutes and at 24, 48, 72 and 96 hours after rFIX dosing (*n* = 4). Blood clotting was monitored on a rotational thromboelastometry (ROTEM; Pentapharm GmbH, Munich, Germany) instrument using the non-activated thromboelastometry (NATEM) reagents. The reaction was initiated by the addition of CaCl_2_ provided with the NATEM reagents from the manufacturer. The coagulation parameters, including clotting time (CT; defined as the time from the initiation of the reaction to an amplitude of 2 mm), clot formation time (CFT; defined from 2–20 mm amplitude) and alpha-angle (defined as the angle of tangent between 2 mm and 20 mm and the curve), were measured [19].

### Efficacy studies in hemophilia B mice

Acute efficacy was studied in the tail clip bleeding model [20,21] with some modifications. Hemophilia B mice (8–16 weeks old) were anesthetized with a mixture of 50 mg/kg ketamine and 0.5 mg/kg dexmedetomidine. Then 5 minutes following the tail vein injection of rFIXFc, rFIX or vehicle solution, the distal 4 mm of the tail was clipped, and the shed blood was collected into 13 mL of warm saline for 30 minutes and quantified gravimetrically (*n* = 20). Linear regression curves of median blood loss and the percentage of protection versus the log (base 10) of dosing concentration were plotted. Mice were considered protected if they bled equal to or less than the mean + 2 standard deviations (SDs) of the blood loss in normal C57BL/6 mice following the same procedure. Prophylactic efficacy was studied in the tail vein transection (TVT) bleeding model as previously reported [16], except that the hemophilia B mice were first injected with rFIXFc 72 hours prior to injury or with rFIX at 24 hours prior to the transection, where the diameter of the tail is approximately 3 mm (*n* = 24 or 34).

## Results

### rFIXFc activation by FXIa or FVIIa/TF

**Fig 1:**
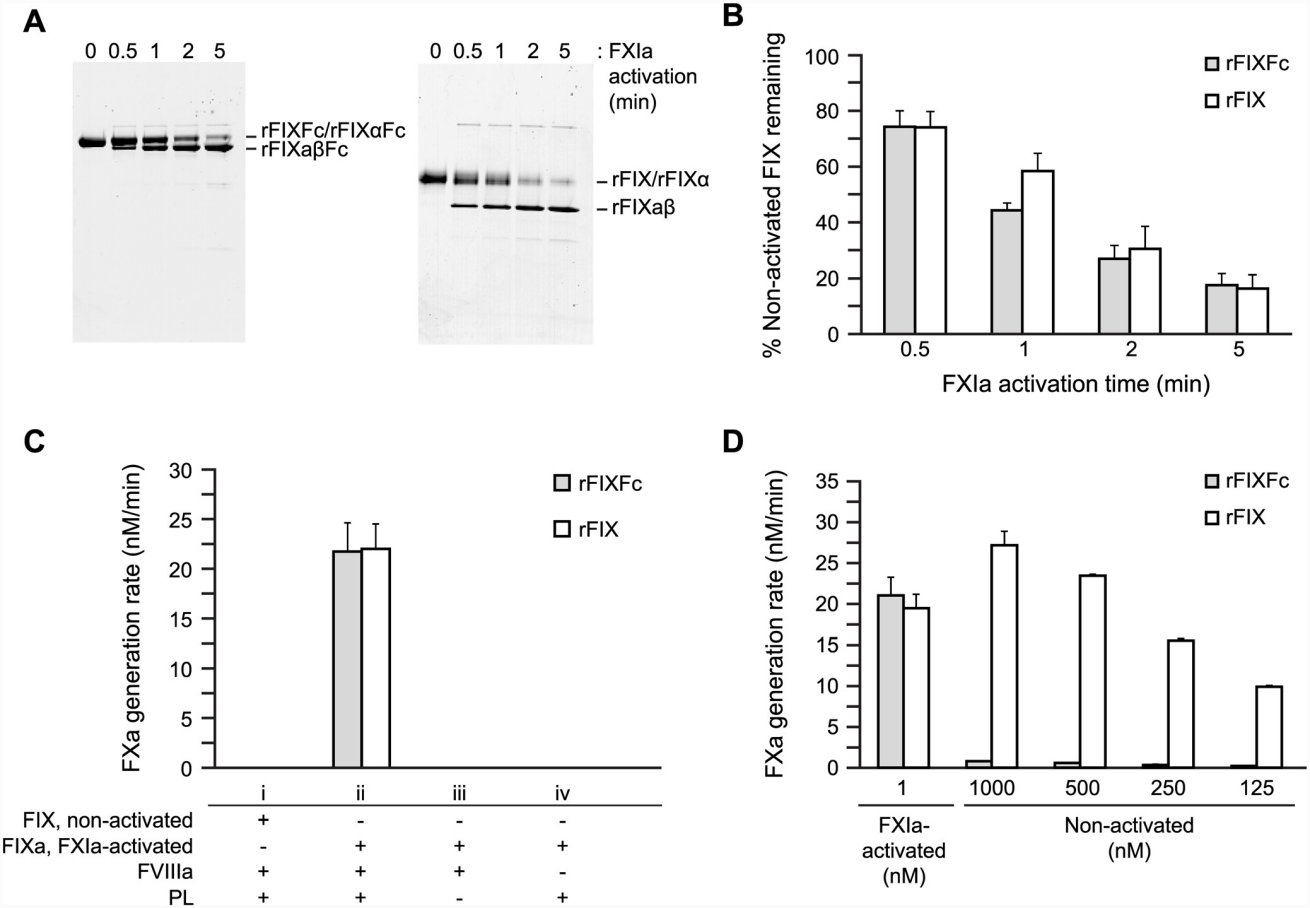
Activation of rFIXFc by FXIa, active Xase complex formation, and rFIXa levels in non-activated rFIXFc. rFIXFc, recombinant factor IX Fc fusion protein; FXIa, activated factor XIa; rFIXa, recombinant activated factor IXa; rFIX, recombinant factor IX; FVIIIa, activated factor VIIIa; PL, phospholipids; FXa, activated factor Xa; SD, standard deviation. (A) Cleavage of rFIXFc by FXIa. rFIXFc (left) or rFIX (right) was activated with FXIa in the presence of calcium chloride at 37°C. Aliquots were collected over 5 minutes at the indicated times and subjected to non-reducing sodium dodecyl sulfate–polyacrylamide gel electrophoresis to resolve the cleaved products. (B) Quantitation of non-activated rFIXFc or rFIX (%) remaining in samples at different times following FXIa activation (mean ± standard deviation (SD); *n* = 3). (C) Formation of active Xase complexes with rFIXaFc requires FVIIIa, PL, and prior activation. Rates of FXa generation were derived from reactions containing rFIXFc or rFIX prior to activation (i) or following activation (ii) by FXIa. Also, rates of FXa generation were calculated for rFIXaFc in the absence of a source of PL (iii, cephalin, rabbit brain extracts) or FVIIIa (iv) (mean ± SD; *n* = 4). (D) Rates of FXa generation (mean ± standard deviation) were determined using rFIXFc or rFIX that were either activated (1 nM) with FXIa or nonactivated (125–1000 nM) to compare the levels of FIXa in rFIXFc and rFIX (*n* = 4).

The activation of FIX into FIXa results from cleavage at two arginine residues (Arg^145^ and Arg^180^) by FXIa or FVIIa/TF. The initial cleavage after Arg^145^ yields the inactive intermediate FIXα, subsequently cleaved at Arg^180^ to the fully active FIXaβ, which is commonly referred to as FIXa [9]. To assess the effect of the Fc moiety on FIX proteolytic activation, rFIXFc and rFIX were cleaved by FXIa, and the generation of the FIXα and FIXaβ forms was evaluated by non-reducing SDS-PAGE (Fig 1A). Cleavage of both molecules yielded the active FIXaβ form, which became predominant over time. The cleavage of both rFIXFc and rFIX followed similar kinetics as apparent by the quantitation of residual FIX at each time point (Fig 1B). At the end of the 5-minute incubation with FXIa, the percent of non-activated FIX remaining was comparable for both rFIXFc (17.6 ± 4.1%) and rFIX (16.3 ± 4.9%; mean ± SD; *n* = 3). Similar results were observed following activation by FVIIa/TF (data not shown). These data reveal that rFIXFc is efficiently activated by FXIa and FVIIa/TF with kinetics similar to that of rFIX.

### rFIXaFc enzymatic activity

The propagation of coagulation depends on the formation of active Xase complexes by FIXa and FVIIIa on the surface of platelets to efficiently convert FX into FXa [22]. To characterize the ability of rFIXaFc to form active Xase complexes, an *in vitro* FXa generation assay was used in the presence of crude brain extracts (cephalin) as source of phospholipids. As expected, in the absence of prior activation, rFIXFc or rFIX resulted in low rates of FXa generation (<0.1 nM/min; Fig 1Ci). Conversely, complexes formed with rFIXaFc or rFIXa exhibited significant rates of FXa conversion (Fig 1Cii) that were dependent on the presence of phospholipids and FVIIIa (Fig 1Ciii and 1Civ, respectively). In addition, both rFIXaFc and rFIXa exhibited similar kinetics when the amidolytic activity was assessed using cleavage of a FIXa chromogenic substrate as readout (data not shown). Taken together, this demonstrates that rFIXFc and rFIX share the same requirements for the formation of active Xase complexes and that the Fc fusion does not affect rFIXFc enzymatic activity.

### rFIXaFc inhibition by AT

**Fig 2:**
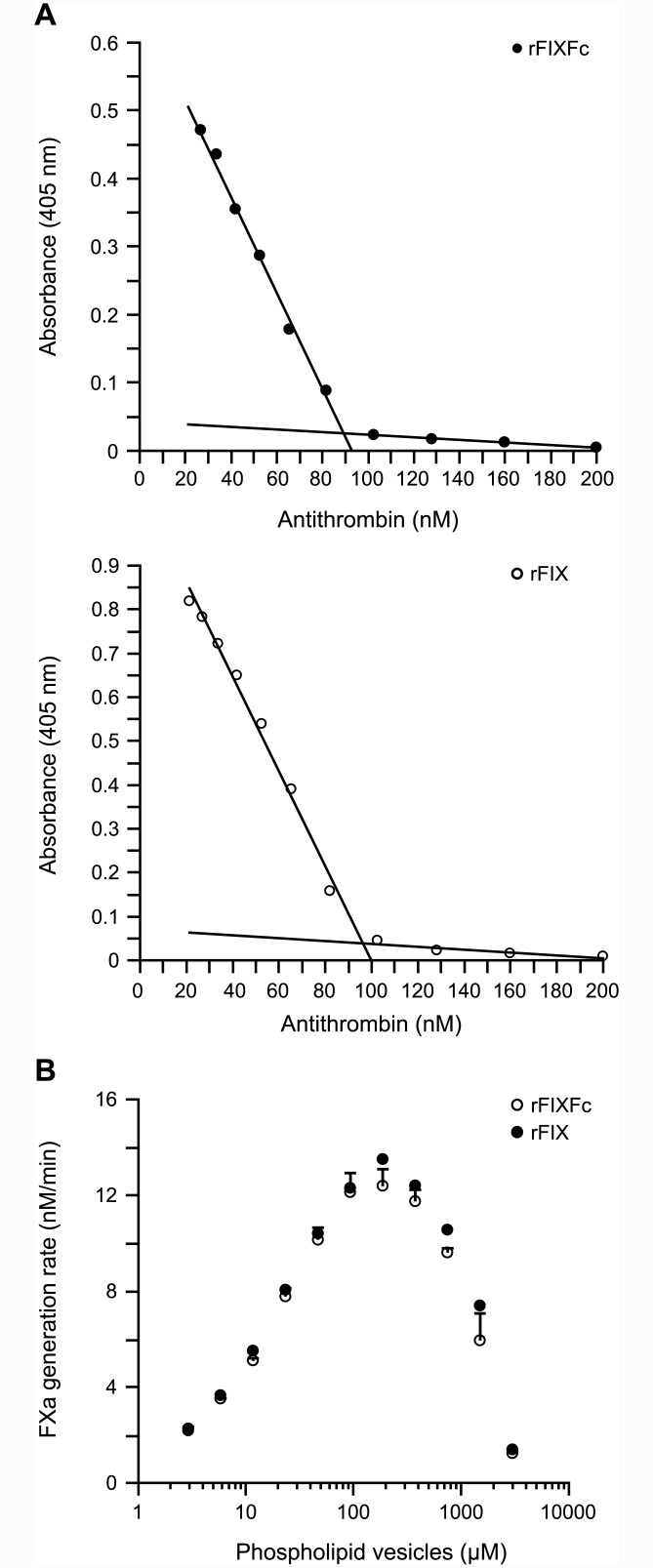
Interaction of rFIXaFc with AT and with phospholipids. rFIXFc, recombinant factor IX Fc fusion protein; AT, antithrombin III; FXIa, activated factor XIa; rFIX, recombinant factor IX; rFIXa, recombinant activated factor IXa; FXa, activated factor Xa; SD, standard deviation; FVIIIa, activated factor VIIIa. (A) Inhibition of rFIXaFc by AT. AT was titrated in 100 nM FXIa-activated rFIXFc or rFIX. The activity of rFIXaFc or rFIXa was monitored using an endpoint FXa generation assay. The amount of FXa generated is reflected in the absorbance at 405 nm as a result of the cleavage of an FXa chromogenic substrate. For each molecule, the data at AT concentrations above or below 100 nM were grouped and fitted (two linear fits per molecule). The point of intersection (inflection point) of both fits was mathematically determined and reflected the concentration of rFIXaFc (89.1% ± 0.55%) and rFIXa (97.2% ± 1.15%) following 5 minutes of FXIa activation (mean ± SD; *n* = 3). (B) Interaction of rFIXaFc with phospholipid vesicles. rFIXaFc or rFIXa was incubated with FVIIIa in the presence of varying concentration of phospholipid vesicles composed of 25% L-α-phosphatidylserine and 75% L-α-phosphatidylcholine to form active Xase complexes. The formed complexes were then evaluated in FXa generation to derive the rates of FXa formation (*n* = 4).

The activity of FIXa is negatively regulated by equimolar binding to the serine protease inhibitor AT in the presence of heparin [23]. To determine the impact of Fc on the regulation of rFIXaFc by AT, the activity of rFIXaFc or rFIXa was determined in the presence of AT using FXa generation as readout. For this, a fixed amount of FXIa-activated rFIXFc or rFIX (100 nM) was incubated with varying AT concentrations (20–200 nM) in the presence of heparin and then assayed for the conversion of FX into FXa. The increasing concentrations of AT inhibited both rFIXaFc and rFIXa activity by correspondingly increasing levels (Fig 2A), and the activity of either molecule was minimal in the presence of molar excess of AT (>100 nM). We conclude that AT inhibition of rFIXaFc is properly maintained.

Because of the 1:1 binding stoichiometry of FIXa and AT, this assay also allows for the determination of actual FIXa concentrations following FXIa (or FVIIa/TF) activation [24]. The concentration of FIXa is reflected by the concentration at which AT inhibition of FIXa is relieved (Fig 2A; inflection points). Using this method, the yields of rFIXaFc and rFIXa following activation by FXIa were derived and shown to be comparable within the assay variation (89.1 ± 0.55% and 97.2 ± 1.15%, respectively; mean ± SD; *n* = 3).

### rFIXaFc interaction with phospholipids

The interaction of FIXa with platelet phospholipid surfaces is necessary for proper hemostasis. To evaluate the effect of Fc on this interaction, the rates of FXa generation were determined for Xase complexes formed by rFIXaFc or rFIXa in the presence of varying concentrations of PS/PC phospholipid vesicles (Fig 2B). As expected, the rates of FXa generation improved with increasing phospholipid vesicle concentrations and peaked at 100 μM to 300 μM for both molecules, after which the rates were reduced similarly for rFIXaFc and rFIXa. We concluded that the interaction of rFIXaFc with phospholipids was not altered.

### Kinetics of the Xase complex formed by rFIXaFc

**Fig 3:**
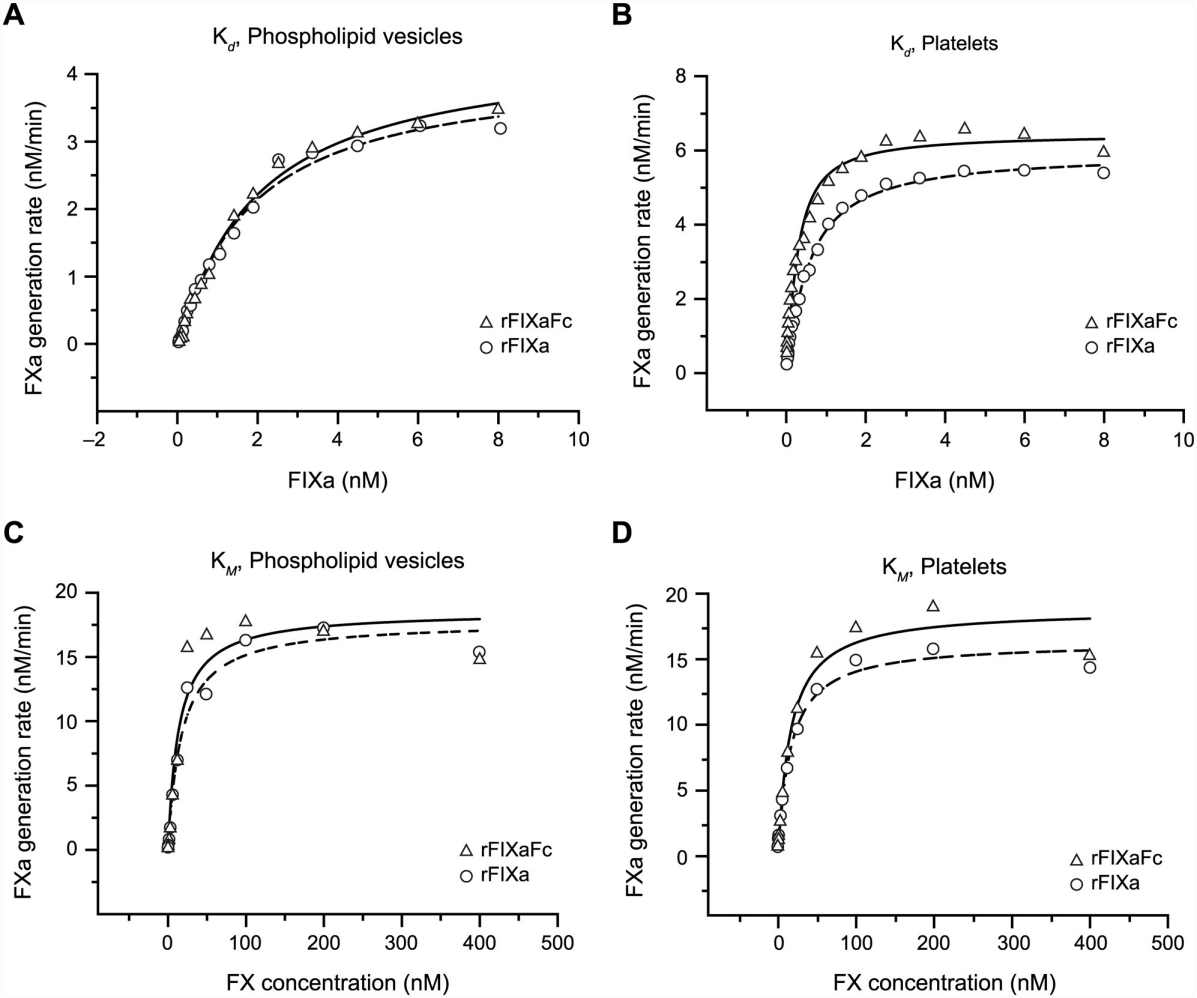
Kinetics of rFIXaFc interaction with FVIIIa and FX. rFIXFc, recombinant factor IX Fc fusion protein; FVIIIa, activated factor VIIIa; FX, factor X; FXa, activated factor Xa; rFIXa, activated recombinant factor IXa; PS, phosphatidylserine; PC, phosphatidylcholine. (A and B) Kinetics of the interaction with FVIIIa. Rates of FXa generation were derived for Xase complexes formed by FVIIIa and varying concentrations of rFIXaFc or rFIXa in the presence of (A) phospholipid vesicles (25% L-α-PS; 75% L-α-PC) or (B) platelets. Binding affinity (K_*d*_) and maximum reaction velocity (V_*max*_) values (Table 1) were derived as described above. (C and D) Kinetics of the interaction with FX. FXa generation rates were derived for Xase complexes formed by FVIIIa and rFIXaFc or rFIXa with varying FX concentrations, in the presence of (C) phospholipid vesicles (25% PS; 75% PC) or (D) platelets, to determine affinity (K_*M*_) and V_*max*_ (Michaelis-Menten equation fit; Tables 2 and 3).

The interaction of FIXa with cofactor VIIIa to form the Xase complex is essential for the improved catalytic activity of FIXa toward FX and subsequent clotting [25]. To evaluate the effect of Fc on this interaction, FXa generation was utilized as readout in the presence of varying rFIXaFc or rFIXa concentrations to derive the FIXa K_*d*_ toward FVIIIa and V_*max*_. These parameters were assessed using cephalin (data not shown), PS/PC vesicles (Fig 3A), or non-activated platelets (Fig 3B) as the source of phospholipids. Both the K_*d*_ of rFIXaFc and rFIXa for FVIIIa and V_*max*_ values were comparable under all conditions tested (Table 1). As expected, the affinities and reaction rates varied equally for both rFIXaFc and rFIXa depending on the source of phospholipids. Therefore, the Fc moiety within rFIXFc did not impact the interaction with cofactor VIIIa.

**Table 1:** Interactions of FXIa-activated rFIXFc and rFIX with FVIIIa in the presence of different phospholipid sources.

Assay components		K_*d*_, nM	V_*max*_, nM/min
Cephalin	rFIXaFc	0.38 ± 0.04	2.5 ± 0.07
Cephalin	rFIXa	0.38 ± 0.05	2.7 ± 0.09
25% PS/75% PC	rFIXaFc	1.74 ± 0.1	4.4 ± 0.1
25% PS/75% PC	rFIXa	1.55 ± 0.1	4.0 ± 0.1
Platelets	rFIXaFc	0.18 ± 0.02	6.5 ± 0.2
Platelets	rFIXa	0.48 ± 0.03	5.9 ± 0.1

PS, L-α-phosphatidylserine; PC, L-α-phosphatidylcholine.

Experiments to determine rFIXaFc and rFIXa kinetics on different phospholipid surfaces were performed (*n* = 4). Concentrations of phospholipids, FX and calcium chloride were kept constant as described in the Methods section. FIXa was added at concentrations ranging from 0.02 nM to 8 nM (platelets) or 0.05 nM to 8 nM (cephalin and phospholipids). Binding affinity (K_*d*_) and maximum reaction velocity (V_*max*_) values (mean ± standard deviation) were obtained by fitting the FXa generation rate data to the K_*d*_ equation as previously described [36].

The K_*M*_ of the Xase complex for FX also was determined to evaluate the impact of Fc on this interaction. In this study, the rates of FXa generation for the complex formed by either rFIXaFc or rFIXa were obtained at varying concentrations of FX in the presence of different sources of phospholipids. First, the K_*M*_ and V_*max*_ of the Xase complex formed by FXIa-activated rFIXFc were assessed and shown to be similar to FXIa-activated rFIX using PS/PC phospholipids (Fig 3C; Table 2) or non-activated platelets (Fig 3D; Table 3). Second, the kinetic parameters were compared for the molecules activated by FXIa or FVIIa/TF (Table 2). In these studies, rFIXaFc and rFIXa exhibited similar kinetic characteristics independent of the activation mode. Finally, these parameters were evaluated using activated or non-activated platelets and were shown to be also similar for rFIXa and rFIXaFc within each condition (Table 3).

**Table 2:** Activity of Xase complex toward FX in the presence of synthetic phospholipids (25% PS/75% PC).

Type of upstream activation	Assay component	K_*M*_, nM	V_*max*_, nM/min
FXIa	rFIXaFc	14.8 ± 3.3	19.0 ± 1.1
FXIa	rFIXa	17.3 ± 2.4	18.0 ± 0.6
FVIIa/TF	rFIXaFc	15.1 ± 3.0	17.8 ± 0.9
FVIIa/TF	rFIXa	14.7 ± 1.7	17.5 ± 0.5

PS, L-α-phosphatidylserine; PC, L-α-phosphatidylcholine.

Experiments were performed (*n* = 4) to determine the kinetics of FX activation by Xase complexes formed using rFIXaFc or rFIXa activated via intrinsic or extrinsic pathways. The concentrations of rFIXaFc or rFIXa, FVIIIa, phospholipids, and calcium chloride were kept constant as described in the Methods section. FX concentrations were varied between 0.39 nM and 400 nM. Affinity (K_*M*_) and maximum reaction velocity (V_*max*_) values (mean ± standard deviation) were obtained by fitting the FXa generation rate data to the K_*M*_ equation as previously described [17].

**Table 3:** Activity of Xase complex toward FX in the presence of non-activated and activated platelets.

Assay components*		K_*M*_, nM	V_*max*_, nM/min
Platelets^†^	rFIXaFc	6.5 ± 1.1	17.4 ± 0.6
Platelets^†^	rFIXa	7.0 ± 1.0	13.2 ± 0.4
Activated platelets^‡^	rFIXaFc	14.9 ± 1.8	31.7 ± 1.0
Activated platelets^‡^	rFIXa	11.4 ± 1.6	21.1 ± 0.7

Experiments were performed to determine the kinetics of FX activation by Xase complexes formed with rFIXaFc or rFIX in the presence of platelets (*n* = 4). The concentrations of rFIXaFc or rFIXa, FVIIIa, and calcium chloride were kept constant as described in the Methods section. FX concentrations were varied between 0.39 nM and 400 nM. Affinity (K_*M*_) and maximum reaction velocity (V_*max*_) values (mean ± standard deviation) were obtained by fitting the FXa generation rate data to the K_*M*_ equation as previously described [17].

*rFIXFc and rFIX were activated with FXIa.

^†^Concentration of non-activated platelets was 2 × 10^8^ cells/mL.

^‡^Platelets (1 × 10^8^ cells/mL) were activated with SFLLRN peptide.

### rFIXaFc levels in rFIXFc

Preparations of BENEFIX^®^ (rFIX) contain residual levels of activated FIX [26]; therefore, we sought to evaluate the levels of rFIXaFc in rFIXFc in comparison with those of rFIXa in rFIX. For this, the FXa generation assay was performed similar to that of Fig 1C in the presence of FVIIIa and cephalin, but with high levels of rFIXFc or rFIX (125–1000 nM) and without prior FXIa or FVIIa/TF activation (Fig 1D). The ensuing rates were compared with a control containing FXIa-activated rFIXaFc or rFIXa (1 nM). The rate of FXa generation by rFIX (500 nM) was comparable to rFIXa (1 nM), whereas FXa generation by rFIXFc at the same concentration (500 nM) was at background level. In general, at every concentration tested, non-activated rFIXFc generated FXa at a rate 35- to 40-fold lower than non-activated rFIX, suggesting that the levels of FIXa in rFIXFc are significantly lower than those in rFIX. These data correlate with the lower levels of FIXa detected in rFIXFc preparations (≤0.013%) compared with rFIX (0.11%) using a FIXa enzyme-linked immunosorbent assay (ELISA) [8,27].

### Thrombin generation profile of rFIXFc

**Fig 4:**
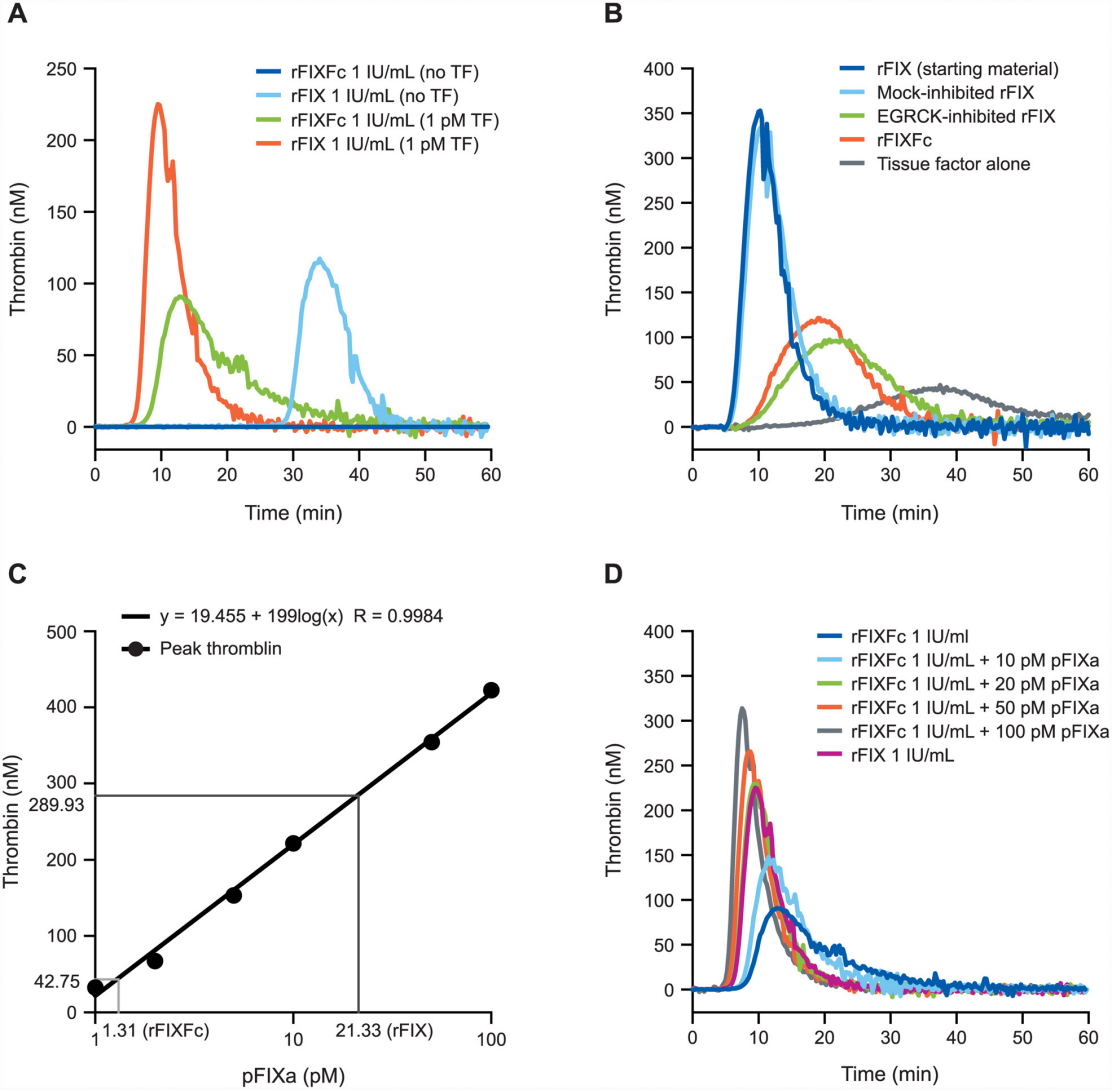
TGA profile of rFIXFc and rFIX. TGA, thrombin generation assay; rFIXFc, recombinant factor IX Fc fusion protein; rFIX, recombinant factor IX; TF, tissue factor; FIXa, activated factor IXa; pFIXa, plasma-derived activated factor IXa; (A) Thrombin generation responses of 1 IU/mL rFIXFc or rFIX triggered with 1 pM TF or no tissue factor controls. (B) Comparison of response to tissue factor triggered-thrombin generation for uninhibited rFIX, rFIX after mock inhibition, rFIX after FIXa inhibition, and rFIXFc. (C) FIXa quantitation standard curve of peak thrombin levels constructed with by spiking increasing concentrations of pFIXa (1, 2, 5, 10, 50 and 100 pM) into FIX-deficient plasma. (D) Thrombin generation responses of 1IU/mL rFIXFc spiked with different amount of pFIXa in comparison with 1 IU/mL rFIX. For each of the panels above, representative results are shown.

To evaluate the thrombin generation potential of rFIXFc relative to rFIX, thrombin generation was triggered by the addition of 1 pM tissue factor (TF) in the presence of 4 μM phospholipids to factor IX-deficient plasma spiked with 1 IU/mL rFIXFc or rFIX, respectively (as determined by the one stage assay; Fig 4A). rFIX generated 2-fold higher peak thrombin and significantly left-shifted the thrombin curve relative to rFIXFc. In a control assay omitting the TF trigger, rFIX demonstrated considerable thrombogenic activity, whereas rFIXFc was essentially inactive (Fig 4A).

Since rFIX was observed to have a markedly higher level of FIXa impurity than rFIXFc (Fig 1D), we further investigated the effect of FIXa impurity on thrombin generation. rFIX was incubated overnight with a serine protease active site blocker, EGR-chloromethyl ketone, and dialyzed by extensive buffer exchange. The FIXa-blocked rFIX showed a very similar thrombin generation profile (ETP, peak thrombin, time course and slope) to rFIXFc (Fig 4B). To quantify the amount of active FIXa in each preparation, a purified, plasma-derived factor IXa (pFIXa) standard curve was constructed by spiking increasing concentrations of factor IXa (0–100 pM) into human factor IX-deficient plasma in the presence of 4 μM phospholipids (5 nM thrombin was added to improve sensitivity). A dose response was observed with a detection limit as low as 0.5 pM pFIXa in FIX-deficient plasma (Fig 4C). rFIX, FIXa-blocked rFIX and rFIXFc of equal potency (1 IU/mL by the one-stage clotting assay) were evaluated and generated thrombin responses comparable to 20 pM, 1 pM and 2 pM pFIXa, respectively, indicating the amount of FIXa present in each FIX product. In a regular thrombin generation assay triggered with 1 pM tissue factor, 1 IU/mL rFIXFc supplemented with 20 pM pFIXa demonstrated an equal peak thrombin and velocity index to 1 IU/mL rFIX (Fig 4D).

### rFIXFc ex vivo activity in hemophilia B mice

**Fig 5:**
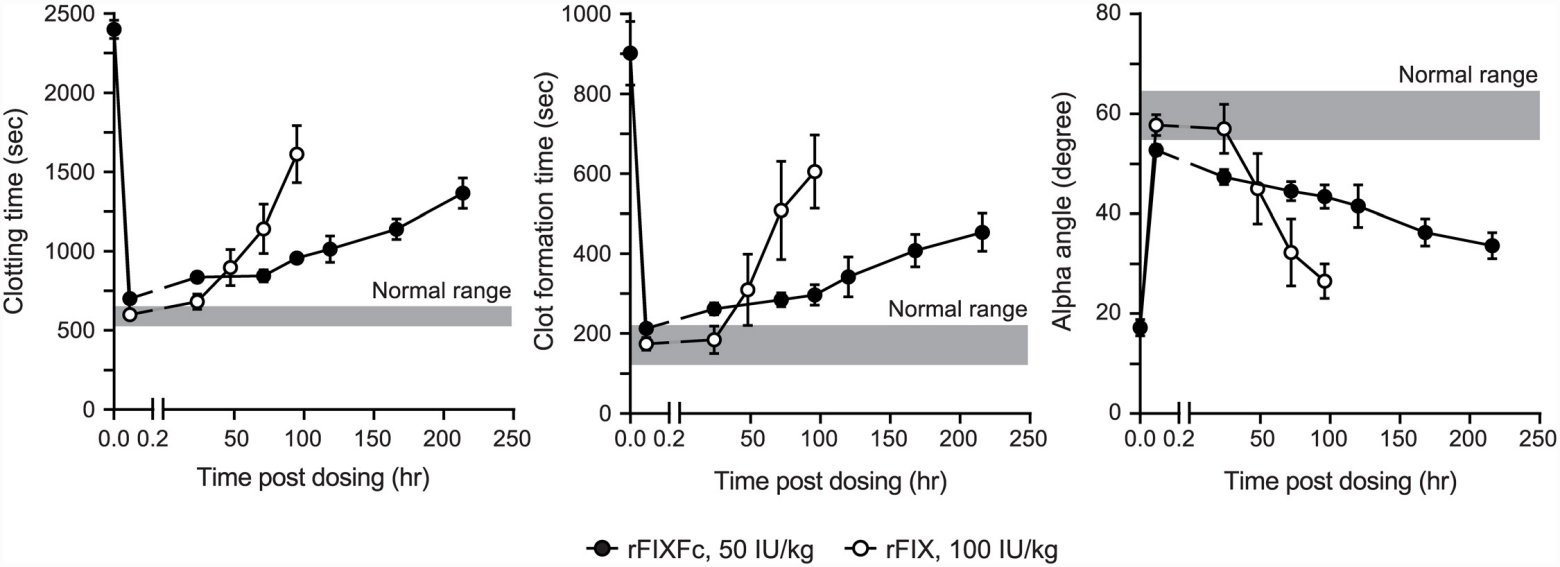
rFIXFc potency and *ex vivo* activity in hemophilia B mice. rFIXFc, recombinant factor IX Fc fusion protein; FIX, factor IX; NATEM, non-activated thromboelastometry; rFIX, recombinant factor IX; CT, clotting time, CFT, clot formulation time; ROTEM, rotational thromboelastometry.*Ex vivo* activity of rFIXFc in hemophilia B mice. Hemophilia B mice were intravenously dosed with equimolar amounts of rFIXFc (50 IU/kg) (*n* = 4 mice/time point) or rFIX (100 IU/kg) (*n* = 8 mice/time point), and whole blood samples collected from these animals at various times were analyzed using the NATEM assay on a ROTEM instrument. Shown are the CT, CFT and alpha-angle means over time.

The half-life of rFIXFc was previously reported to be longer than that of rFIX in multiple species including hemophilia B mice [8]. To determine whether the extension in half-life translates into prolonged activity, mice were intravenously dosed with equimolar levels of rFIXFc or rFIX that translate into 50 IU/kg and 100 IU/kg, respectively, and whole blood samples collected at various times were analyzed using the NATEM assay (Fig 5). At the early time points (5 minutes and 24 hours), the CT, CFT and alpha-angle in samples from mice dosed with rFIXFc or rFIX were comparably improved. However, the improvements in coagulation parameters were sustained over a longer period of time in the presence of rFIXFc despite the 2-fold lower dose relative to rFIX. This demonstrates that the previously observed enhancement in rFIXFc half-life translates into prolonged enhancement in hemostasis in hemophilia B mice.

### rFIXFc efficacy in treating acute bleeds in hemophilia B mice

**Fig 6:**
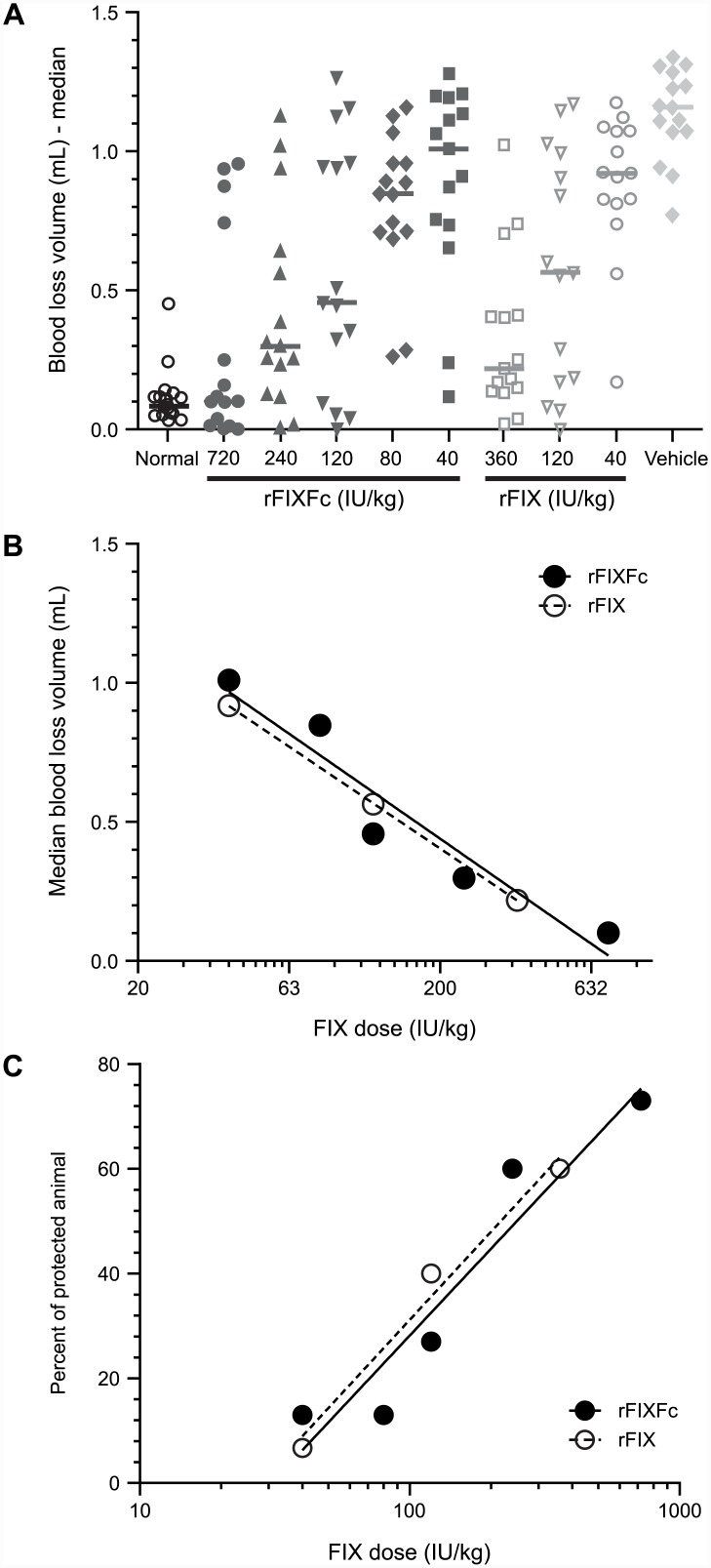
rFIXFc efficacy in treating acute bleeds in hemophilia B mice. rFIXFc, recombinant factor IX Fc fusion protein; rFIX, recombinant factor IX. A tail clip mouse bleeding model was used to evaluate the acute efficacy of rFIXFc. Hemophilia B mice were given rFIXFc (720, 240, 120, 80 or 40 IU/kg, *n* = 15/dose) or rFIX (360, 120 or 40 IU/kg; *n* = 15/dose). (A) Tail clip was performed as described in methods, and the blood loss was quantified. Symbols indicate blood loss from individual mice, horizontal bar median blood loss per group. (B) The linear regression curve of median blood loss versus the log (base 10) of dosing concentration was plotted. Both unpaired *t* test with Welch’s correction and Mann-Whitney test indicated no significant difference between the rFIXFc and rFIX curves (*P* = 0.9315 and 1.0, respectively). (C) The linear regression curve of the percentage of protection versus the log (base 10) of dosing concentration was plotted. Mice were considered protected if they bled ≤ mean + 2 standard deviation of the blood loss in normal C57BL/6 mice following the same procedure. The *t* tests indicated that the two curves derived from rFIXFc and rFIX treatment groups were comparable with *P* values of 0.9385 and 0.8801, respectively.

The efficacy of rFIXFc in treating acute bleeds was evaluated in a tail clip bleeding model. This well-established model involves an arterial injury which mimics severe bleeding episodes in events of trauma and surgeries. It was used as a standard model to evaluate the *in vivo* potency of the therapeutic clotting factors, and because of the high severity of the injury, it requires supraphysiological circulating factor levels to completely resolve bleeds [20]. Using this model, hemophilia B mice were intravenously treated with escalating doses of rFIXFc (40–720 IU/kg) or rFIX (40–360 IU/kg) and compared with vehicle-treated mice. Mice were injured by tail clip 5 minutes post dosing, and the median blood loss and the percentage of protected animals for each group were determined (Fig 6). Both rFIXFc and rFIX treatments significantly reduced blood loss in comparison to the vehicle control (*P* <0.05; unpaired *t* test with Welch’s correction Fig 6A). Further, rFIXFc and rFIX showed comparable dose response in median blood loss (Fig 6A and 6B) and percentage of protection (Fig 6C). Therefore, the results indicate that rFIXFc and rFIX are equally effective in resolving acute bleeds in hemophilia B mice.

### Prolonged prophylactic efficacy of rFIXFc in hemophilia B mice

**Fig 7:**
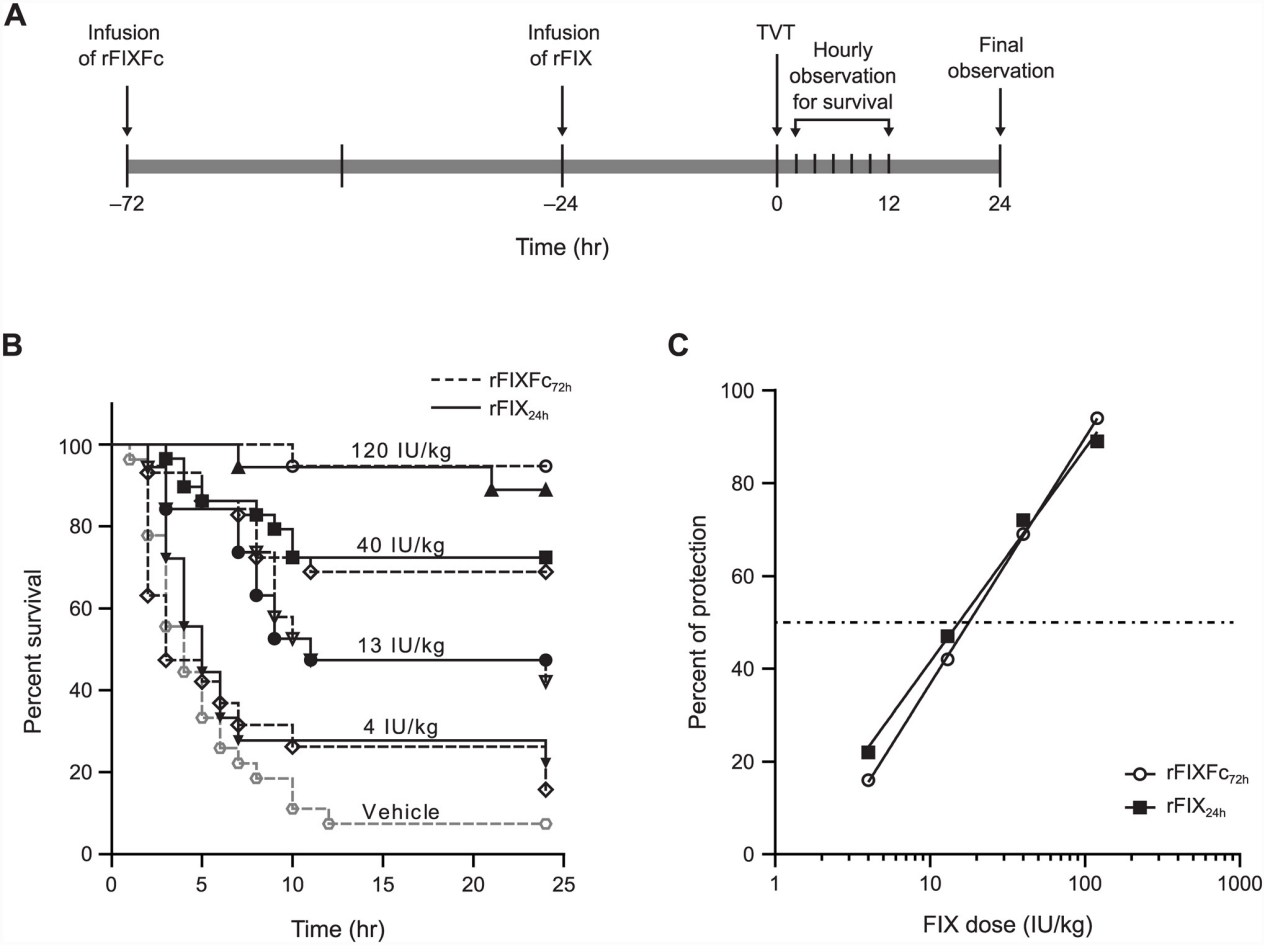
rFIXFc prophylactic efficacy in hemophilia B mice. rFIXFc, recombinant factor IX Fc fusion protein; TVT, tail vein transection; rFIX, recombinant factor IX; rFIXFc_72h_, recombinant factor IX Fc fusion protein dosed 72 hours prior to injury; rFIX_24h_, recombinant factor IX dosed 24 hours prior to injury. (A) TVT study design in hemophilia B mice. Dosing of rFIXFc was performed 72 hours prior to injury, whereas that of rFIX was 24 hours prior to injury. (B) Percent survival following TVT. Mice were dosed with vehicle (grey), rFIXFc_72h_ (black dashed line, open symbols), rFIX_24h_ (black solid line, closed symbols) (120 IU/kg, 40 IU/kg, 13 IU/kg or 4 IU/kg; *n* = 20/dose except for 40 IU/kg dose; *n* = 30). Following tail vein transection (TVT), mice were monitored over 24 hours and the survival curve of time to re-bleed was generated. The survival curves for mice treated with similar doses of rFIXFc_72h_ or rFIX_24h_ were comparable using a log-rank (Mantel-Cox) test (*P* = 0.4886, 0.9203, 0.7574 and 0.5210 for the 4-, 13-, 40- and 120-IU/kg treatment groups, respectively). (C) Dose-response curves at 24 hours following TVT. Survival rates were plotted for both rFIXFc_72h_ (open circles) and rFIX_24h_ (closed squares) treatments. The ED_50_ values were derived for rFIXFc_72h_ treatments (17.8 IU/kg) and rFIX_24h_ (15.4 IU/kg).

To determine whether the 3-fold extension in rFIXFc half-life in animal models translates into prolonged protection from bleeding, the prophylactic efficacy of rFIXFc and rFIX were compared using a tail vein transection (TVT) bleeding model of hemophilia B mice, which is designed to mimic spontaneous bleeding episodes in patients with hemophilia by inducing venous bleeding in a single lateral tail vein. It requires 1% to 5% circulating FIX activity for protection, which is similar to the target trough FIX levels in patients with severe hemophilia B after prophylactic replacement factor treatment [16]. Using this model, mice were treated intravenously with four dose levels of rFIXFc or rFIX (4 IU/kg, 13 IU/kg, 40 IU/kg or 120 IU/kg) and injured at various times post infusion. Mice receiving rFIXFc were injured 72 hours post dosing (dosed with rFIXFc_72h_), whereas those receiving rFIX were injured 24 hours post dosing (dosed with rFIX_24h_) to correlate with the 3-fold extension in rFIXFc half-life (Fig 7A). It was found that comparable doses of rFIXFc_72h_ and rFIX_24h_ result in comparable survival curves (Fig 7B) despite the 3-fold longer delay in time to injury following rFIXFc_72h_ treatment. The effective dose for a 50% response (ED_50_) of rFIXFc_72h_ (17.8 IU/kg) was similar to the ED_50_ of rFIX_24h_ (15.4 IU/kg), indicating a 3-fold increase in prophylactic efficacy of rFIXFc compared with rFIX (Fig 7C).

## Discussion

With the goal of extending the circulating half-life of FIX, a new recombinant fusion molecule, rFIXFc, was molecularly engineered to couple a single molecule of FIX at its C-terminus to the N-terminus of the dimeric Fc domain of human IgG1. The Fc construct was selected to enhance the stability and half-life of rFIX by delaying the degradation of the construct within lysosomes and returning it into circulation [14].

In the present study, the procoagulant activities of rFIXFc were assessed biochemically and compared with rFIX to determine whether the Fc moiety affects rFIXFc function. Specifically, rFIXFc activation, inhibition and interactions with components of Xase complex (phospholipids, platelets, FVIIIa and FX) and its thrombin generation activity were evaluated.

The activation of rFIXFc by FXIa and FVIIa/TF followed kinetics similar to those of rFIX, and the inactivation of rFIXaFc by AT was also found to be comparable to that of rFIXa and correlated with the observed level of inactivation. We note that in the present work, the absorbance levels observed in the AT titration assays with rFIX were higher than those of rFIXFc; however, this is not due to an inability of rFIXaFc to interact with AT, but rather a reflection of the slightly higher levels of activated rFIX in this particular lot and set of experiments (ie, at 60 nM AT, the 37 nM rFIXa generates higher amounts of FXa than the 29 nM rFIXaFc). We have found that in later studies, there were other instances in which rFIXFc was activated to a greater extent than rFIX (S1 Fig) indicating that there was no trend for differential activation and, furthermore, that when the levels of rFIXa and rFIXaFc were normalized using the concentrations determined in the AT studies, the absorbance values reflecting FXa generation were equal (S2 Fig), demonstrating that the differences in absorbance achieved in Fig 2A are due to differences in original concentration. Furthermore, the molecular interactions of rFIXFc with phospholipids, platelets, FVIIIa and FX were all maintained at similar affinities to those of rFIX. Taken together, these results demonstrate that the Fc region does not interfere with the molecular interactions of the FIX moiety in rFIXFc necessary for hemostatic function.

Non-activated rFIXFc preparations were found to have lower intrinsic levels of FIXa activity than did rFIX, which is consistent with previous results using a FIXa ELISA [8] and non-activated partial thromboplastin time assay (data not shown).

The thrombin generation profiles of rFIXFc and rFIX were compared by spiking equal units of rFIX into congenital FIX-deficient plasma. The significant decrease in thrombin generation response of rFIXFc relative to rFIX, considerable thrombogenic activity of rFIX in the absence of tissue factor, and the higher level of FIXa in non-activated rFIX led to the hypothesis that the lower *in vitro* thrombin generation profile of rFIXFc compared with rFIX was due to the presence of excess activated rFIXa in preparations of rFIX. Using a novel and sensitive method of quantitating levels of FIXa (modified thrombin generation assay and careful processing of FIX to remove the FIXa content), we demonstrated that the lower peak thrombin and lengthened time course in the thrombin generation profile for rFIXFc relative to rFIX was caused entirely by the presence of contaminating FIXa in the commercially available rFIX, equivalent to approximately 20 pM pFIXa. We also showed that upon neutralization of the activated rFIXa found in commercially available rFIX, rFIXFc and rFIX had equivalent *in vitro* thrombin generation activity per unit of FIX. Early preparations of plasma-derived FIX often contained small amounts of activated FIX, which caused thrombogenicity. In general, newer replacement FIX products contain lower FIXa levels, and the results shown here demonstrate that this trend is continuing with rFIXFc (eg, FIXa levels of 0.21% ±0.010% with plasma-derived FIX, 0.11% ±0.0019% with rFIX, and <0.013% with rFIXFc [8]). Furthermore, using the Wessler stasis model in rabbits, the thrombogenic activity of rFIXFc was similar to vehicle and less than that of prothrombin complex concentrate and rFIX (BENEFIX^®^) [28]. As FIXa levels have been reported to be correlated with *in vivo* thrombogenicity of high-purity FIX [29], reduced FIXa levels in replacement FIX products may correspond to diminished thrombogenic risk; however, the applicability of this association, which was demonstrated in preclinical models, to clinical experience with commercially available products is unknown.

We have previously observed a difference in specific activity between rFIXFc and rFIX products as assessed by 1-stage clotting (FIX-specific activated partial thromboplastin time-based) assays [8]. On a molar basis, the specific activity of rFIXFc is in the range of 6.5 to 7.1 IU/nmol [27], which is approximately 2-fold lower than rFIX with a specific activity of approximately 12 IU/nmol [8]. The current biochemical characterization of rFIXFc indicates that the difference in specific activity is not attributed to any deficiency in either the activation kinetics of rFIXFc by FXIa or FVIIa/TF, or the molecular interactions within the Xase complex; therefore, the exact mechanism remains under investigation.

The thrombin generation data indicate that on a unit basis, we observe comparable potency to rFIX once the contaminating FIXa has been neutralized. This was also reflected by a similar correction of acute bleeds in hemophilia B mice that received either rFIX or rFIXFc (Fig 6). However, when monitored over time, the improvements in clotting parameters in hemophilia B mice as measured here by ROTEM were sustained longer with rFIXFc than with rFIX. Importantly, this prolongation in rFIXFc activity correlates well with longer efficacy *in vivo*. This was shown by the prolonged protection of hemophilia B mice receiving rFIXFc from TVT injury compared with those receiving rFIX. This prolonged efficacy correlates with the extension of half-life observed for rFIXFc in previous studies [8]. In the phase 3 trial, rFIXFc was associated with a reduction in the frequency of infusions compared with pre-study dosing frequencies, likely due to the half-life extension of rFIXFc coupled with prolonged protection from bleeding [15,30]. Patients with hemophilia have described the frequency of infusions and demanding regimen for prophylaxis therapy with replacement clotting factors as being a considerable burden and a major challenge/barrier to undertaking prophylaxis treatment [31,32]. A reduction in infusion frequency could possibly translate into an improvement in adherence to prophylaxis, as has been demonstrated in studies that show increased compliance with reduced dosing frequency in other chronic diseases [33,34]. Moreover, better adherence to a prophylactic regimen for hemophilia is associated with improved outcomes [35].

In conclusion, rFIXFc was molecularly engineered to provide a long-acting FIX molecule that would reduce the frequency of intravenous injections required for FIX replacement therapy and to improve upon options for the management or prevention of bleeding in patients with hemophilia B. In this study, rFIXFc retained critical procoagulant attributes of rFIX, when assessed in the context of the Xase complex. Importantly, rFIXFc exhibits similar thrombin generation *in vitro* and acute efficacy *in vivo* compared with rFIX; it also exhibits prolonged prophylactic efficacy in animal models of hemophilia B, consistent with the extended half-life shown in previous preclinical studies [8]. These data are consistent with recent publications on the use of rFIXFc in phase 3 clinical studies [15].

## Supporting Information

S1 Fig Interaction of rFIXaFc with antithrombin (non-normalized factor activities). rFIXaFc, activated recombinant factor IX Fc fusion protein; rFIXFc, recombinant factor IX Fc fusion protein; rFIX, recombinant factor IX; rFIXa, activated recombinant FIX; FXa, activated factor X. Inhibition of additional lots of rFIXaFc, rFIXa by antithrombin. Antithrombin was titrated in 100 nM FXIa-activated rFIXFc or rFIX. The activity of rFIXaFc or rFIXa was monitored using an endpoint FXa generation assay. The amount of FXa generated is reflected in the absorbance at 405 nm as a result of the cleavage of an FXa chromogenic substrate.
(EPS)

S2 Fig Interaction of rFIXaFc with antithrombin (normalized factor activities). rFIXaFc, activated recombinant factor IX Fc fusion protein; rFIXFc, recombinant factor IX Fc fusion protein; rFIX, recombinant factor IX; rFIXa, activated recombinant factor IX; FXa, activated factor X. Inhibition of rFIXaFc by antithrombin. rFIX and rFIXFc levels were normalized based on prior active site titration shown in S1 Fig and then used in the assay. Antithrombin was titrated in 100 nM FXIa-activated rFIXFc or rFIX. The activity of rFIXaFc or rFIXa was monitored using an endpoint FXa generation assay. The amount of FXa generated is reflected in the absorbance at 405 nm as a result of the cleavage of an FXa chromogenic substrate.
(EPS)

S1 Text Supplemental Methods. (DOCX)

S2 Text ARRIVE Guidelines Checklist. (PDF)
