# Supplementary material for: Recombinant Factor IX Fc Fusion Protein Maintains Full Procoagulant Properties and Exhibits Prolonged Efficacy in Hemophilia B Mice
Source: PLoS One. 2016 Feb 3;11(2):e0148255. doi: 10.1371/journal.pone.0148255 (PMC4740463; doi:10.1371/journal.pone.0148255)
Supplement: S1 Text — (DOCX) [file pone.0148255.s003.docx]

**S1 Text. Supplemental Methods**

**Activation of rFIXFc by FXIa or FVIIa/TF**

For activation by FXIa, rFIXFc or rFIX was diluted to 3 µM in 50 mM Tris (Tris(hydroxymethyl)aminomethane) (pH 7.4), 100 mM sodium chloride (NaCl), 10 mM calcium chloride (CaCl_2_) and 0.2% bovine serum albumin (BSA) and incubated with 30 nM FXIa at 37ºC for 5 minutes. The activated mixtures were placed on ice following the 5-minute incubation, aliquoted in pre-chilled tubes and stored at –20ºC. For the activation by FVIIa/TF, FVIIa (10 nM) and lipidated TF (3 nM) were diluted in 50 mM Tris (pH 7.4), 100 mM NaCl, 10 mM CaCl_2_ and 0.2% BSA and incubated at 37ºC for 15 minutes. rFIXFc or rFIX was then added (3 µM) with CaCl_2_) to maintain the concentration at 10 mM. The mixtures were incubated at 37ºC for 1 hour, after which the activated mixtures were placed on ice, aliquoted in pre-chilled tubes, and stored at –20ºC. The activation of rFIXFc and rFIX by FXIa or FVIIa/TF was verified by sodium dodecyl sulfate–polyacrylamide gel electrophoresis (SDS-PAGE) analysis.

**Inhibition of rFIXaFc by AT**

The assay was conducted in 50 mM Tris (pH 7.4), 100 mM NaCl, 10 mM CaCl_2_ and 0.2% BSA. FXIa-activated rFIX (rFIXaFc or rFIXa) was diluted to 200 nM and heparin was added at a concentration of 10 IU/mL. AT was serially diluted separately at concentrations ranging from 400 nM to 43 nM. Equal volumes (25 µL) of rFIXaFc or rFIXa and AT dilutions were combined and the mixtures were incubated at room temperature (RT) for 30 minutes. The residual activity of rFIXaFc or rFIXa was assessed toward FX in an FXa generation assay. For this, FX (200 nM) and cephalin (5 µL/reaction, resuspended according to manufacturer’s directions) were added to the AT/heparin/rFIXaFc or rFIXa mixtures and incubated at RT for 20 minutes. The FXa generation assay was conducted purposely in the absence of FVIIIa to control reaction rates and facilitate data collection on a measureable timescale. Following the incubation with FX, an FXa chromogenic substrate (Pefachrome FXa 5523; Centerchem, Norwalk, CT, USA) was added (0.5 mM) for 30 minutes at RT before reading the endpoint absorbances at 405 nm.

**Formation of the Xase complex**

Full-length recombinant FVIII was incubated at RT for 5 minutes with 2 nM α-thrombin to generate FVIIIa. Hirudin (5 nM) was added to FVIIIa to neutralise α-thrombin and prevent FVIIIa inactivation along with 1 nM rFIXaFc or rFIXa in the presence of 5 mM CaCl_2_ and 5 µL cephalin (resuspended according to the manufacturer directions) as the phospholipid source. The FVIIIa-FIXa (Xase) complex was allowed to form for 10 minutes, and its activity was assayed by adding 100 nM FX and 0.5 mM FXa chromogenic substrate (Pefachrome FXa 5523). Absorbance at 405 nm was recorded and FXa generation rate was determined as previously described [17]. This assay was run in parallel under three other conditions, each with one component missing (FIXa, phospholipids or FVIIIa).

**Affinity of rFIXaFc for FVIIIa**

The affinity of rFIXaFc and rFIXa for FVIIIa was determined using three different phospholipid sources (25% PS/75% PC, cephalin or platelets). In each case, rFIXaFc or rFIXa was diluted (0.02-8 nM for platelets or 0.05-8 nM for phospholipids and cephalin) in 50 mM Tris (pH 7.4), 100 mM NaCl, 5 mM CaCl_2_ and 0.2% BSA. A mix containing hirudin (10 nM) and either phospholipids (100 μM), cephalin (5 µL/reaction upon resuspension of cephalin based on manufacturer’s directions) or platelets (as will be described) was added to each rFIXaFc or rFIXa dilution. This was followed by the addition of FVIIIa (0.2 nM) previously activated for 5 minutes at RT with thrombin (2 nM). The FVIIIa/FIXa mixture was incubated for 10 minutes at RT to allow for the formation of the Xase complex. Following this incubation, FX (100 nM) and FXa chromogenic substrate (0.5 mM), or fluorogenic substrate (125 μM) in the case of platelets, were then added to the reaction mix and the absorbance or fluorescence intensities were monitored over time. FXa generation rates were determined and data were fitted to determine binding affinity (K*_d_*) and maximum reaction velocity (V*_max_*) as previously described [17,36].
